# Trends in child maltreatment in Germany: comparison of two representative population-based studies

**DOI:** 10.1186/s13034-018-0232-5

**Published:** 2018-05-25

**Authors:** Andreas Witt, Heide Glaesmer, Andreas Jud, Paul L. Plener, Elmar Brähler, Rebecca C. Brown, Jörg M. Fegert

**Affiliations:** 1 Department of Child and Adolescent Psychiatry/Psychotherapy, University of Ulm, Steinhövelstr. 5, 89075 Ulm, Germany; 2 Department of Medical Psychology and Medical Sociology, University of Leipzig, Leipzig, Germany; 3 Department of Psychosomatic Medicine and Psychotherapy, University Medical Center of Johannes Gutenberg University Mainz, Mainz, Germany

**Keywords:** Child maltreatment, Child abuse and neglect, Trends, Epidemiology

## Abstract

**Background:**

Child maltreatment of all types is a serious concern for society, and it is important to monitor trends in incidence in order to inform child welfare agencies and policy-makers about emerging issues. In Germany, however, information on such trends is limited: apart from official sources, the only published study is a comparison of surveys conducted in 1992 and 2011 that had focused primarily on sexual abuse. The present study is the first to look at more recent trends and to examine other types of maltreatment as well.

**Methods:**

We compared the datasets of two population-based nationwide surveys, one conducted in 2010 (N = 2504) and the other in 2016 (N = 2510). Both had used identical methodology. Participants aged 14 years and older had been selected randomly using the Kish selection grid method, and information about childhood experiences of abuse had been solicited using the Childhood Trauma Questionnaire.

**Results:**

The overall percentage of respondents who reported having experienced at least one type of child maltreatment decreased over the 6 years, from 35.3% in 2010 to 31.0% in 2016; however, the percentages who reported multiple types of maltreatment remained stable. The decrease in any type of maltreatment was mainly driven by fewer reports of physical neglect, which was likelier to be reported by older respondents who had experienced privation during the (post-) war years and whose representation was lower in the later survey. There was a significant increase over time in the prevalence of emotional abuse, with respondents aged 26–45 years reporting higher rates of this type of maltreatment. The prevalence rates of other types of maltreatment remained unchanged. All effect sizes were very small.

**Conclusions:**

At present, the systems in place in Germany for monitoring the occurrence of child maltreatment are insufficient. While this study contributes to a better understanding, more information is needed, particularly on populations that have been excluded or underrepresented in previous research efforts. As has been done elsewhere, large databases should be set up, using identical methodologies and definitions, in order to accurately assess trends over time in different types of abuse and neglect.

## Background

The issue of child maltreatment is a serious public health concern that calls for research into strategies for prevention, interventions, and epidemiological developments. Research in this field is complicated as no uniform definition of child maltreatment exists. However, the Centers of Disease Control in the US have proposed a definition based on a multi-professional consensus process that has gained much attention [1]. Child maltreatment is defined as any act or series of acts of commission or omission by a parent or other caregiver that results in harm, potential for harm, or threat of harm to a child (p. 11 [1]). Child maltreatment is further subdivided into acts of commission including sexual, emotional and physical abuse and acts of omission including failure to provide and failure to supervise. An important aspect of research in this field is reliable monitoring of trends in prevalence, as the findings serve to inform health care providers, social services providers, and policy-makers about emerging issues. However, debate over the data on trends has long been controversial [2–4].

Among the first to report a decline in rates of sexual abuse were Finkelhor et al. [4–7] who collected evidence from multiple sources in the United States, including population-based surveys, agency surveys and administrative data sets. The reliability of the declining trend is supported by the similarity in decreasing rates obtained from agencies and self-reports. Apart from sexual abuse, Finkelhor et al. also presented evidence on trends for other forms of violence against children [4, 6, 8]; however, the data sets for information on these other types of maltreatment are considerably smaller. For physical abuse findings from different sources are mixed [3, 4, 6] and there is no clear evidence for a decline in physical abuse in the US. With respect to neglect, Finkelhor and Jones [4] concluded that prevalence remained stable between 1990 and 2003, but there is a paucity of data on this form of maltreatment, and little is known about trends. In another review, Gilbert et al. [9] examined trends in six developed countries, assessing more severe types of child maltreatment. Despite the implementation of national policy initiatives designed to reduce the problem, these researchers found no consistent evidence of a change in prevalence rates. However, as they were looking at only the most severe types of maltreatment, their results may not contradict the findings of a decline in overall rates child sexual abuse. In sum, at least for the US, there is reliable evidence for a decline in child sexual abuse, while more evidence on the trends for other types of maltreatment is needed.

In Germany, the topic of child maltreatment, especially sexual abuse has gained public and political awareness after the disclosure in early 2010 of severe and extensive sexual abuse of children in institutions [10–12]. These disclosures triggered an intense media coverage and broad public debate, leading politicians to take action. As a result a Round Table with the aim to develop recommendations for prevention and intervention and an Independent Commissioner for child sexual abuse issues were installed. The scandal lead to a range of legislative changes [13] with implications for data collection and the practice. For example the coding of child maltreatment in the medical sector became possible. Additionally, a new data collection on initial risk assessments by local child welfare agencies was introduced and professional competence in the risk assessment was strengthened. Also, in 2012 a federal law came into force that for the first time provided a uniform nationwide regulation on confidentiality for professionals.

**Fig. 1:**
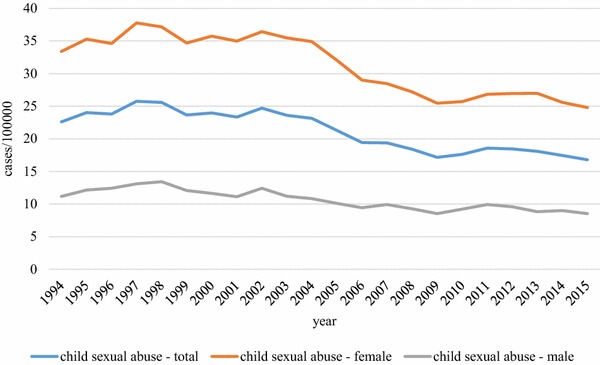
Official German police crime statistics of child sexual abuse (Penal Code § 176) between 1994 and 2015

The debate about trends in prevalence of child maltreatment in Germany has focused on sexual abuse, and only very limited data on that are available. The major sources of information are criminal statistics databases [14], but these are limited to reported cases that meet a legal definition so in terms of numbers represent only a small amount and has been referred to as the tip of the iceberg [15]. The numbers of cases of sexual abuse officially included in crime statistics between 1994 and 2015 are presented in Fig. 1. The apparent decline may be due to an actual decrease in prevalence rates, but may also be due at least in part to a change in reporting procedures.

Apart from data on sexual abuse, the police databases have maintained information on physical abuse of children since 2001, when an official ban on corporal punishment was introduced, but they do not collect data on other types of child maltreatment such as neglect and emotional abuse. The only source for these is the child welfare sector, which provides reports from local agencies on emergency measures [16] and initial risk assessments [17]. In contrast to the crime statistics, agency reports show a constant rise in substantiated cases of child endangerment, rising from 16,875 cases in 2011 to 20,806 cases in 2015, an increase of 19%. However, the data collection processes are new, and this increase might be due to changed approaches in reporting and implementation.

Prior to now, only one population-based study has been conducted on trends in child maltreatment in Germany, and that looked primarily at sexual abuse. Stadler et al. [18] compared two representative national surveys, the first conducted in 1992 [19] and the second in 2011 [18], and carried out analyses both within and between samples. Adult respondents of various ages were asked for retrospective information on whether they had been sexually abused prior to the age of 14, and conclusions about trends were drawn by comparing the rates of different age cohorts within each sample and of the same age cohorts between the samples. Within-sample comparisons in the earlier survey found that women in the oldest age cohort (30–39 years) reported higher rates of childhood sexual abuse than did those in the younger cohorts (21–29 and 16–20 years) [19]. Findings for the later survey were similar, with the prevalence reported by women in the oldest cohort about 3 times higher compared to the youngest cohort. The results for men were similar but less pronounced. In the comparisons across surveys, a significant decrease in rates was seen overall: in the 1992 survey, childhood sexual abuse was reported by 9.1% of women and 2.9% of men, while in 2011, the rates were 6.7 and 1.4%, respectively. This result remained stable when different age cohorts were contrasted between the two studies. The authors concluded that for both sexes, the experience of sexual abuse was significantly less prevalent among younger respondents, indicating a decline over time. However, this study has been criticized for the limited comparability of the two surveys, as different methodologies had been used to carry them out. Further, a range of biases may have affected the prevalence rates, such as cohort-specific appraisals and definitions. Additionally, actual increases in the rates in younger age cohorts may not be clearly determined as recollection and memory biases may not be differentiated from real changes. Accordingly, these results have to be interpreted with caution.

In summary, data on trends in child maltreatment in Germany are very limited [7, 20, 21], with the only evidence coming from the single study described above which examined only sexual abuse. The World Health Organization stresses the need for a comprehensive and reliable national data collection system to monitor trends in all types of child maltreatment in order to evaluate measures, interventions, and prevention programs [7, 9, 22], and in fact Germany has been criticized by the United Nations Committee on the Rights of the Child for having insufficient information in this field [23]. The aim of the present study was to assess trends in numerous types of child maltreatment based on two more recent representative population surveys conducted 6 years apart.

## Methods

### Procedure

Two population-based surveys were conducted in 2010 and 2016, using identical procedures. In both, the services of a demographics research institute (USUMA, Berlin, Germany) were used to select a representative sample of respondents, employing a random route approach. Starting with a specific address, every third household was approached by a researcher, and residents of the identified households were asked whether they would be willing to complete a set of questionnaires. For multi-person households, one person was randomly selected to be the respondent, using the Kish selection grid technique. Participants had to be at least 14 years of age and have sufficient German language skills. Prospective subjects were told only that the study was about psychological health and well-being (only one of the questionnaires dealt with child abuse), child maltreatment as a specific topic of the study was not mentioned explicitly beforehand. Afterwards, informed consent was obtained from those who indicated a willingness to take part. Main reasons for non-participation were failure to contact anyone in the residence (2010: after three attempts: 11.1%, 2016: after four attempts: 14.9%), refusal by the individual who answered the door to have anyone in the household participate in the study (2010: 12.8%; 2016: 15.3%), failure to contact the randomly selected household member (2010: after three attempts: 2.8%, 2016: after four attempts: 2.3%) and refusal by the selected member to participate (2010: 15.6%; 2016: 14.7%). Of those who participated, only 25 respondents did not complete the CTQ.

Anonymity in responses was guaranteed. After obtaining sociodemographic information through a face-to-face interview, the researcher gave the participant the questionnaires, along with an envelope in which to seal them when done, and then left the room but remained nearby in case any assistance was required. The completed questionnaires were linked to the respondent’s demographic data, but did not contain name, address, or any other identifying information.

The study was conducted in accordance with the Declaration of Helsinki, and fulfilled the ethical guidelines of the International Code of Marketing and Social Research Practice of the International Chamber of Commerce and of the European Society of Opinion and Marketing Research. Prior to being carried out, it was approved by the Ethics Committee of the Medical Faculty of the University of Leipzig.

### Measures

The sociodemographic questionnaire solicited information on age, gender, citizenship, geographical area (rural vs. urban), educational and occupational status, and partnership status.

Different types of child maltreatment were assessed using the 28-item brief version of the Childhood Trauma Questionnaire (CTQ) [24, 25], a widely used instrument that solicits information about five types of maltreatment: emotional, physical, and sexual abuse, and physical and emotional neglect. An additional scale assesses whether participants tend to minimize experiences in their own childhood. The psychometric properties of the German version of the CTQ have been demonstrated by Klinitzke et al. [24] and Wingenfeld et al. [26], with internal consistency ranging between *α *= .62 and *α *= .96 for the subscales. The scale for physical neglect has however been criticized for its low reliability [27]. The intra-class coefficient for an interval of 6 weeks is .77 for the overall scale and between .58 and .81 for the subscales. For each subscale, severity scores are categorized as “none to minimal”, “minimal to moderate”, “moderate to severe”, or “severe to extreme”, using the severity coding of Bernstein et al. [25]. In this study, maltreatment was considered to have occurred if it was classified as at least “moderate to severe”.

### Statistical analyses

Analyses were conducted using SPSS version 21. Severity and prevalence of maltreatment were summarized using descriptive statistics; prevalence rates between the samples were compared using χ^2^ tests and t-tests; and trends were analyzed using χ^2^ tests. For the analyses of trends, each sample was categorized into four age cohorts: 14–25, 26–45, 46–65, and 66 years and older. To assess the interrelatedness of different types of maltreatment, Pearson correlations between different types of maltreatment for both samples were calculated. To account for possible biases due to non-responses and disproportionate distribution of certain demographic characteristics (age, gender, and area of residence), a weighting method was applied to adjust for these biases, and prevalence rates based on the weighted ratings were calculated.

## Results

Detailed results of the first survey are available from Häuser et al. [27]. The results of the comparison of the two surveys are provided below. A total of 5014 participants took part.

### Participants

In the 2010 survey, a total of 4902 households were approached of which 2504 (51%) agreed to take part, while in 2016, 4902 households were approached of which 2510 (51%) took part. The main reasons for non-participation were failure to contact anyone in the residence after a specified number of attempts (11.1% in the first survey, 14.9% in the second), failure to contact the randomly selected household member (2.8 and 2.3%, respectively), refusal by the individual who answered the door to have anyone in the household participate in the study (12.8 and 15.3%, respectively), and refusal by the selected member to participate (15.6 and 14.7%, respectively).

Detailed demographic characteristics for both samples are presented in Table 1. In the 2010 sample, 53.2% of respondents were female, mean age was 50.6 years (SD = 18.6), and 3.7% were did not hold German citizenship; in the 2016 sample, 53.3% were female, mean age was 48.4 years (SD = 18.2), and 3.2% were born outside of Germany. The age difference between the samples reached significance (*t *= 4.4, *p *< .001, *d *= .12). Both samples were representative of the German population with regard to gender and age, but participants not holding German citizenship were underrepresented [28].

**Table 1:** Sociodemographic characteristics

	2010			2016		
	Total (N = 2504)	Female (N = 1331)	Male (N = 1173)	Total (N = 2510)	Female (N = 1339)	Male (N = 1171)
Age (years)						
Mean (standard deviation)	50.6 (18.6)	51.6 (18.6)	49.6 (18.5)	48.4 (18.2)	48.9 (18.1)	47.8 (18.4)
Range	14–90	14–90	14–90	14–94	14–94	14–93
Living with partner						
Yes	1517 (60.6%)	769 (57.8)	748 (63.8%)	1370 (55%)	719 (54%)	651 (56.2%)
No	987 (39.4%)	562 (42.2%)	425 (36.2%)	1119 (45%)	612 (46%)	507 (43.8%)
Citizenship						
German	2411 (96.6%)	1298 (97.5%)	1113 (94.9%)	2429 (96.8%)	1303 (97.3%)	1126 (96.2%)
Not German	93 (3.7%)	33 (2.5%)	60 (5.1%)	81 (3.2%)	36 (2.7%)	45 (3.8%)
Geographical area^a^						
Rural	1034 (41.3%)	543 (40.8%)	491 (41.9%)	1026 (40.9%)	548 (40.9)	478 (40.8%)
Urban	1470 (58.7%)	788 (59.2%)	682 (58.1%)	1484 (59.1%)	791 (59.1%)	693 (59.2%)
Occupational status						
Full-time	954 (38.1%)	354 (26.6%)	600 (51.2%)	1074 (42.8%)	407 (30.4%)	667 (57%)
Part-time	206 (8.2%)	189 (14.2%)	17 (1.4%)	281 (11.2%)	246 (18.4%)	35 (3%)
Hourly	46 (1.8%)	42 (3.2%)	4 (.3%)	60 (2.4%)	54 (4%)	6 (.5%)
Federal volunteer service/parental leave	12 (.5%)	11 (.8%)	1 (.1%)	25 (1%)	22 (1.6%)	3 (.3%)
Unemployed	152 (6.1%)	78 (5.9%)	74 (6.3%)	131 (5.2%)	64 (4.8%)	67 (5.7%)
Retiree	793 (31.7%)	412 (31%)	381 (32.5%)	638 (25.4%)	368 (27.5%)	270 (23.1%)
Homemaker	179 (7.1%)	175 (13.1%)	4 (.3%)	79 (3.1%)	77 (5.8%)	2 (.2%)
In training	29 (1.2%)	10 (.8%)	19 (1.6%)	42 (1.7%)	21 (1.6%)	21 (1.8%)
Student	133 (5.3)	60 (4.5%)	73 (6.2%)	161 (6.4%)	70 (5.2%)	91 (7.8%)
Equivalised disposable income (€/month)						
< 1250	649 (26.9)	379 (29.6)	270 (23.7)	435 (18)	264 (20.5)	170 (15.2)
1250–2500	1583 (65.5)	803 (62.8)	780 (68.5)	1579 (65.7)	841 (65.3)	738 (66)
> 2500	185 (7.7)	97 (7.6)	88 (7.7)	392 (16.3)	182 (14.1)	210 (18.8)

^a^Participants were counted as “urban” if they were living in a community with more than 20,000 inhabitants

### Prevalence and severity of child maltreatment

Since the age difference between the samples was significant, separate analyses were conducted on weighted prevalence rates. The unweighted and weighted prevalence rates for each type of maltreatment are presented in Tables 1 and 2, respectively. The results were similar, indicating that any bias due to age difference was minimal (see Table 3).

**Table 2:** Prevalence rates of different types of maltreatment

	2010			2016			2010 vs. 2016 (total) $\chi^2$ (p)	$\varphi$
	Total (N = 2504)	Female (N = 1331)	Male (N = 1173)	Total (N = 2510)	Female (N = 1339)	Male (N = 1171)		
At least one type of maltreatment^a^	881 (35.3%)	476 (35.9%)	405 (34.6%)	772 (31%)	431 (32.6%)	341 (29.3%)	10.11 (.001)*	.045
Multiple types of maltreatment^b^	342 (13.7%)	192 (14.5%)	150 (12.9%)	347 (14%)	208 (15.8)	139 (12%)	.08 (.77)	–
Emotional abuse	115 (4.6%)	71 (5.3%)	44 (3.8%)	163 (6.5%)	115 (8.7%)	48 (4.1%)	8.88 (.003)*	.042
Physical abuse	139 (5.6%)	75 (5.7%)	64 (5.5%)	167 (6.7%)	86 (6.5%)	81 (6.9%)	2.75 (.097)	–
Sexual abuse	156 (6.2%)	112 (8.4%)	44 (3.7%)	190 (7.6%)	150 (11.9%)	40 (3.4%)	3.65 (.056)	.027
Emotional neglect	348 (13.9%)	177 (13.4%)	171 (14.6%)	332 (13.3%)	186 (14%)	146 (12.5%)	.44 (.51)	–
Physical neglect	719 (28.8%)	378 (28.5%)	341 (29.1%)	562 (22.5%)	292 (22%)	270 (23.1%)	25.71 (< .001)*	.072

* p < .05

^a^Participants were counted if they reported at least one type of maltreatment with a severity score of “moderate to severe” or higher

^b^Participants were counted if they reported at least two types of maltreatment with a severity score of “moderate to severe” or higher

**Table 3:** Weighted prevalence rates for different types of maltreatment

	2010			2016		
	Total %	Female %	Male %	Total %	Female %	Male %
At least one type of maltreatment^a^	33.8	34.7	32.8	31.9	33.9	29.7
Multiple types of maltreatment^b^	13.2	14.3	12.1	14	15.8	12.1
Emotional abuse	4.3	5.1	3.4	6.1	8.2	3.9
Physical abuse	5.3	5.6	5	6.9	6.8	7.1
Sexual abuse	6.2	8.4	3.8	7.6	11.5	3.6
Emotional neglect	13.2	12.7	13.8	12.9	13.7	12.1
Physical neglect	27.8	27.9	27.7	23.7	23.8	23.6

^a^Participants were counted if they reported at least one type of maltreatment with a severity score of “moderate to severe” or higher

^b^Participants were counted if they reported at least two types of maltreatment with a severity score of “moderate to severe” or higher

The percentage of respondents who reported having experienced at least one form of maltreatment as a child was higher in the 2010 sample (35.3%) than in the 2016 sample (31.0%), a difference that reached significance (*χ*^*2*^= 10.1, *p *= .001). The overall percentages of respondents who reported more than one type of maltreatment did not differ significantly (13.7% in 2010 vs. 14.0% in 2016; *χ*^*2*^= .08, *p *= .77). With regard to specific types of maltreatment, rates of reported emotional abuse increased significantly over time (4.6% in 2010 vs. 6.5% in 2016; *χ*^*2*^= 8.9, *p *= .003), while rates of reported physical neglect decreased significantly (28.8% in 2010 vs. 22.5% in 2016; *χ*^*2*^= 25.7; *p *< .001). For other types of maltreatment, the rates did not differ significantly between the samples. Of those respondents who reported any maltreatment, the rates for having experienced one type were 21.5% in 2010 vs. 16.8% in 2016; for two types, 8.2% vs. 7.0%; for three types, 2.2% vs. 3.7%; for four types, 2.3% vs. 2.1%; and for five types, 1.0% vs. 1.2%.

The severity scores for each type of maltreatment are presented in Table 4.

**Table 4:** Distribution of severity of different types of maltreatment

	Emotional abuse		Physical abuse		Sexual abuse		Emotional neglect		Physical neglect	
	2010	2016	2010	2016	2010	2016	2010	2016	2010	2016
None to minimal	2123 (85%)	2027 (81.3%)	2198 (88%)	2185 (87.1%)	2186 (87.4%)	2148 (86.1%)	1259 (50.5%)	1486 (59.5%)	1288 (51.6%)	1452 (58.2%)
Minimal to moderate	259 (10.4%)	302 (12.1%)	162 (6.5%)	145 (5.8%)	158 (6.3%)	158 (6.3%)	888 (35.6%)	678 (27.2%)	491 (19.6%)	482 (19.3%)
Moderate to severe	75 (3%)	98 (3.9%)	70 (2.8%)	83 (3.3%)	109 (4.4%)	133 (5.3%)	184 (7.3%)	155 (6.2%)	450 (18%)	336 (13.5%)
Severe to extreme	40 (1.6%)	65 (2.6%)	69 (2.8%)	84 (3.4%)	47 (1.9%)	57 (2.3%)	164 (6.6%)	177 (7.1%)	269 (10.7%)	226 (9.1%)

### Interrelatedness of different types of maltreatment

Table 5 shows the significant intercorrelations of all types of maltreatment (all correlations *p *< .001). The pattern of interrelatedness is comparable in both samples, with the highest correlations seen between physical and emotional abuse and between physical and emotional neglect. The correlations were in the moderate to high range, except for that between sexual abuse and physical and emotional neglect, which was in the low range.

**Table 5:** Correlations between different types of maltreatment

	Emotional abuse		Physical abuse		Sexual abuse		Emotional neglect		Physical neglect	
	2010	2016	2010	2016	2010	2016	2010	2016	2010	2016
Emotional abuse										
2010	1		.672		.493		.459		.479	
2016		1		.633		.461		.545		.426
Physical abuse										
2010			1		.538		.375		.491	
2016				1		.472		.448		.470
Sexual abuse										
2010					1		.245		.326	
2016						1		.274		.273
Emotional neglect										
2010							1		.587	
2016								1		.635
Physical neglect										
2010									1	
2016										1

### Comparison of prevalence rates in age cohorts between and within samples

The comparison of prevalence rates in the four age cohorts is presented in Table 6. There was a significant difference in the rates of physical neglect, with the highest prevalence in the oldest age cohort. The within-sample comparisons show that in both samples, there were significant differences in rates for physical abuse among the oldest three age cohorts. The comparison also shows a significant difference in rates of emotional abuse in the age cohorts of 26- to 45-year-olds.

**Table 6:** Comparison of prevalence rates in four age cohorts within and between samples

	14–25, N(%)^a^	26–45, N(%)^a^	46–65, N(%)^a^	> 65, N(%)^a^	$\chi^2$ (*p*)
Emotional abuse					
2010	11 (4.2%)	*36 (4.8%)*	46 (5.5%)	22 (3.4%)	3.9 (.27)
2016	17 (5.2%)	*62 (8%)*	58 (6.5%)	26 (5.1%)	5.4 (.15)
$\chi^2$ (*p*)	.33 (.57)	*6.7 (.009)* ^b^	.75 (.39)	2.2 (.14)	
$\varphi$	–	.066	–	–	
Physical abuse					
2010	11 (4.2%)	31 (4.1%)	47 (5.6%)	50 (7.7%)	9.5 (.023)
2016	20 (6.2%)	45 (5.8%)	62 (6.9%)	40 (7.9%)	2.3 (.52)
$\chi^2$ (*p*)	1.1 (.3)	2.4 (.12)	1.3 (.26)	.01 (.92)	
$\varphi$	–	–	–	–	
Sexual abuse					
2010	13 (5%)	44 (5.8%)	61 (7.3%)	38 (5.8%)	2.7 (.44)
2016	16 (5%)	58 (7.5%)	82 (9.2%)	34 (6.7%)	7 (.07)
$\chi^2$ (*p*)	.0 (.98)	1.7 (.19)	2 (.16)	.35 (.55)	
$\varphi$	–	–	–	–	
Emotional neglect					
2010	25 (9.6%)	111 (14.7%)	124 (14.9%)	88 (13.6%)	5.1 (.17)
2016	33 (10.2%)	96 (12.4%)	135 (15.2%)	68 (13.4%)	5.9 (.11)
$\chi^2$ (*p*)	.05 (.8)	1.7 (.19)	.03 (.87)	.01 (.92)	
$\varphi$	–	–	–	–	
Physical neglect					
2010	32 (12.3%)	*172 (22.8%)*	*215 (25.7%)*	*300 (46.3%)*	*148.4 (< .001)*
2016	41 (12.6%)	*133 (17.2%)*	*193 (21.6%)*	*195 (38.9%)*	*104.2 (< .001)*
$\chi^2$ (*p*)	.02 (.9)	*7.4 (.007)*	*4 (.044)*	*7.3 (.007)*	
$\varphi$	–	.069	.048	.079	

^a^N (%) represents the number and percentage of participants in each age cohort in each sample who reported having experienced this type of maltreatment

^b^Italic type was used to indicate significant differences between the sample

## Discussion

Studies on trends in child maltreatment that used information obtained from regularly updated databases in the United States have found a marked decrease in rates of sexual and to some extent physical abuse. The results, especially on sexual abuse, seem to represent a real decline rather than being attributable to changes in reporting or disclosure behavior or to methodological artifacts [5]. In Germany, no such databases exist, and the sources of available information are limited and difficult to compare. The present study is the first to examine trends in multiple types of child maltreatment in this country, based on the findings of two relatively recent surveys that were conducted 6 years apart. This is especially relevant, since Germany has seen measures being taken after the abuse scandals in 2010.

The overall prevalence rates reported in the first survey, which have been described in detail by Häuser et al. [27], were largely replicated in the second one, thereby supporting the reliability of the reported prevalence rates in the general population and for the use of the CTQ as a reliable measure. Both samples provided evidence that various types of child maltreatment, especially physical and emotional neglect, are common, but that the prevalence of different types varies considerably. The comparison between the two samples revealed a decrease over the 6 years in the overall rate of having experienced at least one type of maltreatment, but no difference in having experienced more than one type. Therefore, the overall rate might have decreased, while combinations and probably more severe types might have remained stable. However, with percentages of 31 and 35.3%, an alarming number of people are affected and results underline the significance of child maltreatment as a major health problems. The mean age of respondents was older in the earlier survey (50.6 vs. 48.4 years), but as there was no substantial difference between weighted and unweighted data, the effects of the age difference appear to be negligible.

In line with other findings in the literature [29], about 14% of participants in both samples reported having experienced multiple types of maltreatment, with similar patterns of co-occurrences. The co-occurrence of multiple types of maltreatment was rather the rule than the exception. The size of the correlations between the different types of maltreatment ranged from small (.24), seen between emotional neglect and sexual abuse, to large (.67), seen between emotional abuse and physical abuse. The interrelatedness was highest between physical and emotional abuse and between physical and emotional neglect; less so between emotional or physical abuse and neglect; and least between sexual abuse and either type of neglect.

In general, for the youngest age cohorts (aged 14–25 years), comparisons between the samples showed no significant difference for any type of maltreatment, supporting the conclusion that on a population level, prevalence rates had remained stable. Since the samples were spaced only 6 years apart, none of the respondents who were older than 25 at the time of the later survey would have been children at the time of the earlier one, so trends over this time period would be revealed only in the youngest age cohorts.

With respect to sexual abuse, the finding of no significant differences in rates differs from the finding of Stadler et al. [8], who noted a sharp decrease in the rates reported by younger respondents. That study, however, was based on a much longer time span, with the second survey conducted almost 20 years after the first [19], whereas the present study addresses a much shorter time period and provides more recent data. The Stadler findings were consistent with official crime statistics [14], which similarly reported a decrease of cases over the same period of time. However, as in the present study, Stadler et al. obtained their information about childhood experiences retrospectively from an adult sample, which makes comparisons with the incidences reported by agencies difficult. Additionally, the study has been criticized because of the limited comparability of the two surveys due to different methodologies.

In the current study, one of the findings from the comparison of age cohorts within each sample was that in both surveys, reports of physical neglect were more prevalent among older respondents, especially those aged over 65, and steadily decreased towards the youngest cohort; while between-sample comparisons showed that such reports were significantly lower in the each of the three oldest cohorts in 2016 compared to their 2010 counterparts. However, this decrease could be due to a smaller representation in 2016 of the generation who had experienced privation in the (post-) war years. The psychometric properties of the physical neglect scale of the CTQ have been criticized for lack of reliability [24, 26], and assenting to statements such as “I did not have enough to eat” may in some cases be indicative of general food shortages rather than of parental neglect. Additionally, the effect sizes were small [30]. Therefore, this finding should be interpreted with caution.

Another finding of interest was a significant increase in reports of emotional abuse, primarily attributable to a higher rate among women aged 26–45 years. This increase does not seem to be attributable to higher exposure rates, as participants in this age cohort in the later survey would not have been children at the time of the earlier survey (the youngest would have been 20 years old). It is more likely due to a growing awareness of this type of abuse and consequently a higher level of disclosure. Especially after the abuse scandals in 2010 in Germany, there was an increase in public and political awareness of the topic [10]. Media campaigns have been launched and a range of measures have been taken (see “Background” section). Even though the medial and political focus was on sexual abuse, legislative changes were not targeted exclusively on sexual abuse, but also other types of maltreatment. Also reliable data on different types of maltreatment from the child welfare sector is now available. This might have also increased the general awareness. Therefore, it is likely that awareness for all types of maltreatment, especially those that have rarely been in the focus before, such as emotional abuse has increased. Another explanation might be that the increase is due to normal statistical variations between the samples.

The prevalence rate reported here is still considerably lower compared to those found for Europe [20] and worldwide [31]. This type of maltreatment is difficult to assess, and therefore has not been in the focus of researchers or of the public as strongly as have other types, such as sexual abuse. Comparisons within and between the samples of rates of the other types of maltreatment—physical abuse, sexual abuse, and emotional neglect—revealed no significant differences, indicating that they remained stable over the 6 years.

The findings of the present study differ from the official data available from the criminal justice and child welfare systems. However, those sources only capture cases that have been brought to the attention of authorities, and thus account for only a fraction of the real total. Further, their findings are mixed, as they are based on different objectives and different focuses. Crime statistics on child maltreatment involving either sexual abuse or physical abuse, which have been maintained by police since 2001, show a decrease of about 10% from 2010 to 2015 [14, 32], but include only those cases that meet legal definitions, and do not capture any cases involving physical neglect, emotional abuse, or emotional neglect. In contrast to the decline seen in the police statistics, data from the child welfare sector show a rise in all types of maltreatment: between 2012 and 2015, local child welfare agencies reported increases of 13.0% in cases of confirmed physical abuse and of 24.6% in confirmed cases of neglect [17, 33], and between 2010 and 2015, they reported increases of about 17% in the number of emergency measures taken in response to physical abuse and about 12% on emergency measures taken in response to neglect [16, 34]. In sum, the findings of data sets from different administrative sources show trends in different directions. However, because of their different aims and scopes, their results are not necessarily in contradiction to each other or to the findings from the population surveys. It could be that cases are becoming less severe, thus leading to a reduction in the number that meet the criteria for criminal charges; also, there may have been a shift in directing cases to the attention of child welfare authorities rather than the courts, as a better alternative to prosecution. Especially, after the abuse scandal in 2010 measures for early intervention and legislation, clarifying when to notify child protection, investigating and providing support to the families, have been implemented (see “Background” section). Germany is rather family services oriented as in contrast to many Anglo-saxon countries that are rather child protection oriented [9], this may explain some of the discrepancies. Additionally, as in contrast to most of the other European countries there is no mandatory reporting in Germany. Finally, in the US, data show that short-term increases in rates may also due to changes in the law or the implementation of new data acquisition programs [35], and those effects could be a factor here as well.

In summary, even though the numbers found in the present studies are smaller in comparison to meta-analyses [20, 31, 36–38] the percentages of about one-third of the general population that has experienced at least one type of maltreatment is alarming and underline the significance of child maltreatment as a major public health problem. The measures that have been taken after the abuse scandals in 2010 do not seem to have impacted the prevalence rate substantially. However, such measures need time until an impact on the population level can be observed. For example corporal punishment has been legally banned in 2000. In 2009, only 31% were aware of this ban [39]. Still, in 2016 percentages of positive attitudes towards the use of corporal punishment was high, given the ban in 2000 [40]. But studies have shown that knowledge about the ban of corporal punishment has increased after the act has been initiated [41, 42] and that broad media campaigns are needed to disseminate such changes [39]. Albeit, such broad information campaigns, as seen after the abuse scandals in 2010, may also affect prevalence rates of reported child maltreatment. Increases, as observed for emotional abuse may reflect a changed understanding and cultural climate, making the recollection more salient.

## Limitations

In interpreting the results, a range of limitations has to be considered. First, 6 years is a relatively short period in which to identify trends in child maltreatment on a population level, especially when it involves adults being queried about events they experienced in childhood. In Germany, a range of measures, including changes in legislation, were taken after the abuse scandals of 2010, and it will be some time before their full effects are visible at a population level. Since in even this brief time some changes were observable in several subgroups and age cohorts, more detailed monitoring, including incidence data, is necessary in order to gain a better understanding of trends in maltreatment.

Second, the occurrence of child maltreatment was assessed retrospectively and, as is true for all self-report retrospective studies, the results may have been affected by recollection biases. Moreover, the reliability for the physical neglect subscale is low (see “Methods” section); findings for this subscale have to be interpreted with caution. In any case, surveys about childhood experiences in adult populations can only provide information on how rates of maltreatment have changed over generations. To be able to draw conclusions about current trends, larger assessments that are done exclusively on children and adolescents, such as school surveys, are needed.

Third, with respect to representativeness, sample size could have been a limiting factor: the combined sample size of 5014, while not small, is low in comparison to other samples, such as those of the Criminological Research Institute of Lower Saxony [19, 43]. Further, the random route approach used has the effect of systematically excluding people in special housing programs, meaning that high-risk populations, such as residents of child welfare institutions and people with certain disabilities, might have been underrepresented. Rates of child maltreatment have been shown to be considerably higher in such subgroups in comparison to the general population [44]. There was also underrepresentation of people who did not hold German citizenship; and, because data were acquired using questionnaires that respondents had to complete on their own, individuals with insufficient German language skills were excluded.

Finally, the demographic data indicated that the two samples differed on several variables, not just age as described above, but also occupational status and equivalised disposable income, and these differences may have had an impact on the results. However, it is unrealistic with this sample size to achieve complete equivalence at two different points in time, as demographic variables change over the years. In any case, the effect of the age difference was found to be very small, and was likely negligible as the comparison of the weighted and unweighted prevalence rates indicated no significant differences. However, research highlights, some types of maltreatment, e.g. neglect are linked to certain demographic characteristics [45, 46]. Therefore, differences in sociodemographic characteristics, such as socio-economic status might have impacted the results and should be borne in mind when interpreting the results.

## Conclusions

The findings of this study indicate that experiences of some forms of maltreatment in childhood are common in the general German population. Generally, most of the rates of maltreatment remained stable over the last 6 years. Germany has been criticized by the United Nations Committee on the Rights of the Child for collecting insufficient data on this problem, and needs to do more. Large databases, similar to what exists in the US, should be set up, and ongoing monitoring systems need to be designed that track the occurrence not only of sexual abuse but other types of maltreatment as well, using consistent methodologies and definitions in order to accurately assess trends over time. Data need to be obtained from samples of children and adolescents, not just retrospective data from adults who may have been abused in their past; and survey systems should be expanded to ensure the inclusion of high-risk populations such as people living in institutions and/or who are not fluent in the German language. Implementation of these measures should provide policy-makers and those working in the child welfare section with the information they need in order to properly evaluate the effectiveness of prevention and intervention measures.
